# Supplementary figures and images for: Comparison of vaginal microbiota between women with inflammatory bowel disease and healthy controls
Source: PLoS One. 2023 Nov 29;18(11):e0284709. doi: 10.1371/journal.pone.0284709 (PMC10686494; doi:10.1371/journal.pone.0284709)

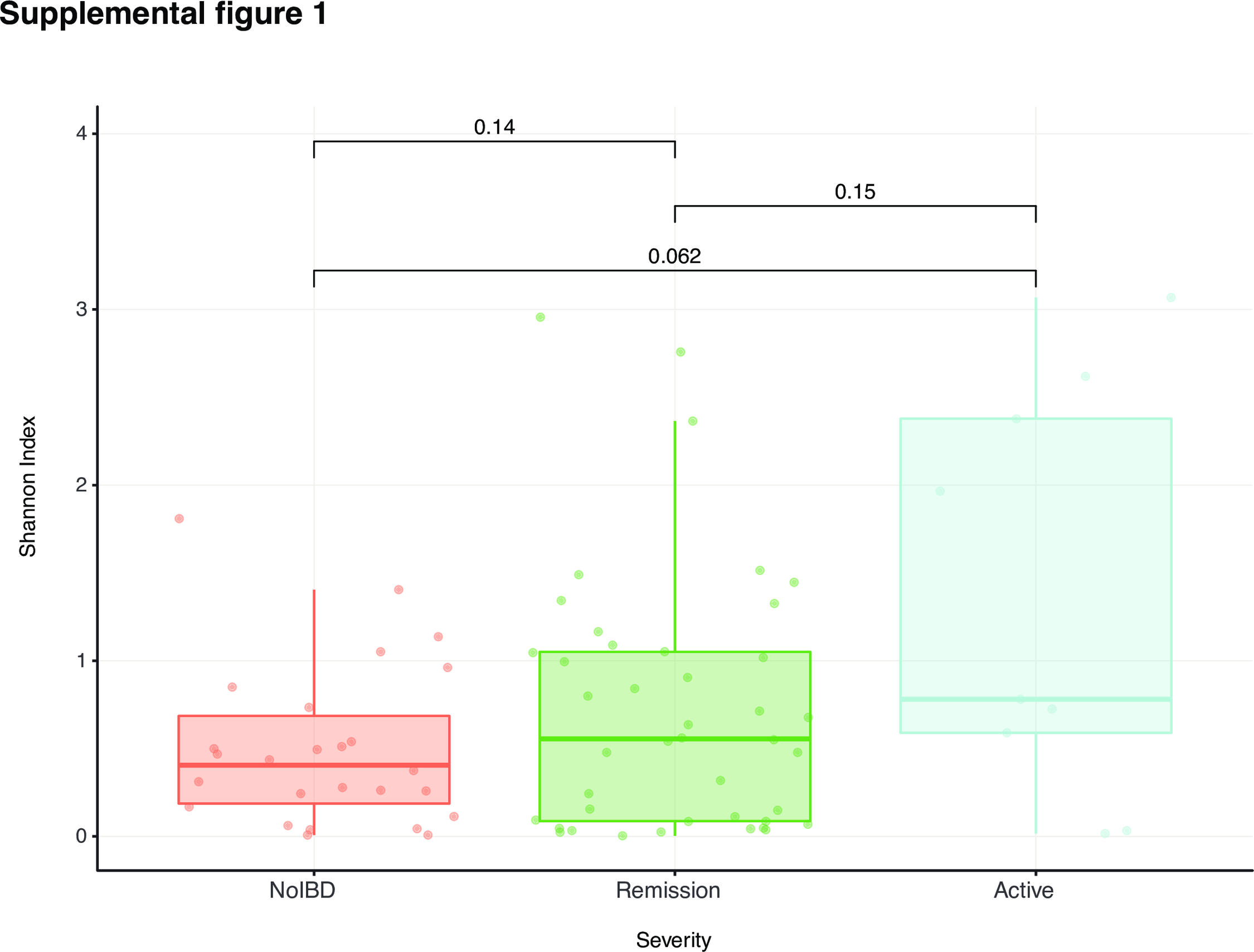

Supplement: S1 Fig — Shannon Diversity Index was used to compare alpha diversity by symptom severity. Gastrointestinal symptom severity was assigned using either the Harvey Bradshaw Index (Crohn’s) or Simple Clinical Colitis Assessment (Ulcerative Colitis). For this analysis a single random sample was selected for each participant (n = 80). (TIF) [file pone.0284709.s001.tif]
